# Periodontal treatment on patients with cardiovascular disease: Systematic review and meta-analysis

**DOI:** 10.4317/medoral.22725

**Published:** 2018-11-21

**Authors:** Elisabet Roca-Millan, Beatriz González-Navarro, Maria del Mar Sabater-Recolons, Antonio Marí-Roig, Enric Jané-Salas, José López-López

**Affiliations:** 1 DDS, Master’s student in Oral Medicine, Surgery and Implantology, Faculty of Medicine and Health Sciences (Dentistry), University of Barcelona, L’Hospitalet de Llobregat, Barcelona, Spain; 2 Department of Odontostomatology. Faculty of Medicine and Health Sciences (Dentistry), University of Barcelona // Oral Health and Masticatory System Group (Bellvitge Biomedical Research Institute) IDIBELL. University of Barcelona, L’Hospitalet de Llobregat, Barcelona, Spain; 3 Department of Odontostomatology, Faculty of Medicine and Health Sciences (Dentistry), University of Barcelona, L’Hospitalet de Llobregat, Barcelona, Spain; 4 Specialist in Maxillofacial Surgery, Head of Department of Maxillofacial Surgery, University Hospital of Bellvitge. Catalonia, Spain. // Oral Health and Masticatory System Group (Bellvitge Biomedical Research Institute) IDIBELL. University of Barcelona, L’Hospitalet de Llobregat, Barcelona, Spain; 5 Full Professor, Department of Odontostomatology. Faculty of Medicine and Health Sciences (Dentistry) - Medical manager and Head of the medical-surgical area of Odontological Hospital University of Barcelona - University of Barcelona // Oral Health and Masticatory System Group (Bellvitge Biomedical Research Institute) IDIBELL. University of Barcelona, L’Hospitalet de Llobregat, Barcelona, Spain

## Abstract

**Background:**

Atherosclerotic cardiovascular disease is the main cause of mortality in developed countries. It is a chronic and systemic inflammatory disease with a multifactorial etiology. Periodontal disease is one of the many factors that contribute to its development.

**Objective:**

To analyze the effects of periodontal treatment on cardiovascular risk parameters in patients with atherosclerotic cardiovascular disease.

**Material and Methods:**

A systematic research was conducted in the Pubmed/Medline databases for clinical trials published up to and including the year 2017.

**Results:**

Ten articles were included for analysis. Periodontal treatment reduced C-reactive protein levels (77.8% of clinical trials), tumor necrosis factor-alpha (66.7%), interleukin-6 (100%) and leukocytes (50%). Fibrinogen levels also improved considerably (66.7%). Effects on lipid parameters were more limited, whereby only oxidized low density lipoprotein and very low density lipoprotein cholesterol decreased significantly. Meta-analysis showed a statistically significant decreased in C-reactive protein and leukocytes values when patients were submitted to non-surgical periodontal treatment in contrast to receiving no treatment at all (mean difference 1.199 mg/L, 95% confidence interval: 1.100-1.299, *p*<0.001; and mean difference 0,79 g/L, 95% confidence interval: 0.717-0.879, *p*<0.001, respectively).

**Conclusions:**

Periodontal treatment has a beneficial effect on some of the biochemical parameters considered to represent cardiovascular risk. Further randomized clinical trials are necessary, with longer follow-up periods including regular periodic monitoring, in order to determine the extent of the impact of periodontal treatment.

** Key words:**Periodontal disease, cardiovascular disease, atherosclerosis, periodontal therapy, periodontal treatment.

## Introduction

Atherosclerotic cardiovascular disease (ACVD) constitutes a group of chronic inflammatory pathologies that include ischemic heart disease, ischemic cerebrovascular disease, and peripheral arterial disease (1,2). It is the main cause of morbidity and mortality in industrialized countries, and of death in the world (1-3). The classic risk factors, such as hypercholesterolemia, hypertension, and smoking, only explain half the deaths of patients with this group of pathologies, and half of severe cardiovascular events (1,3). During the last 10 years, numerous published studies have investigated the relationship between ACVD and periodontal disease, because of the inflammatory basis that they share (1-7). However, research has not reached sufficiently certain conclusions to establish a causal relation between ACVD and periodontal disease. Nevertheless, on the basis of the information acquired to date, the potential positive effect of periodontal treatment on ACVD – if this can be confirmed – could constitute a new preventative/therapeutic tool (3).

There is clear scientific evidence for the important role played by inflammation in the development of ACVD (2). Epidemiological studies support the association between periodontal disease and systemic inflammation (8,9) and various meta-analyses have identified a higher risk of developing cardiovascular disease among periodontal patients (10-14). However, the effect of periodontal treatment on the atherosclerotic profile and the prevention of future cardiovascular events is not so clear (3,15). Analyzing the biochemical parameters considered cardiovascular risk factors can assess the impact of treatment. These can be grouped as inflammatory markers (C-reactive protein (CRP), tumor necrosis factor alpha (TNF-α), interleukin-6 (IL-6) and leukocytes), hemostatic or thrombotic markers, and lipid profile parameters (6).

In this context, the present systematic review was based on the following PICO question: P: periodontal treatment; I: of patients with ACVD; C: compared with non-treatment of periodontal disease; O: improvement to cardiovascular parameters.

## Material and Methods

A literature search was conducted in the Pubmed database with no limitations placed on publication date. The following key search terms were applied: “Cardiovascular Disease” AND “Periodontal Treatment”; “Cardiovascular Disease” AND “Periodontal Therapy”; “Atherosclerosis” AND “Periodontal Treatment”; “Atherosclerosis” AND “Periodontal Therapy”; “Heart Disease” AND “Periodontal Therapy”; “Heart Disease” AND “Periodontal Treatment”; “Inflammatory Markers” AND “Periodontal Treatment”; and “Inflammatory Markers” AND “Periodontal Therapy”.

The titles and abstracts of all the articles located were read to select clinical trials analyzing the effect of periodontal treatment on the different clinical and biochemical parameters related to ACVD. Works not written in English or Spanish were discarded, as were animal studies. Literature reviews and meta-analyses were also excluded, as well as clinical trials that assessed the effect of periodontal treatment on cardiovascular risk in patients with diabetes or metabolic syndrome. The full text was read in case of any doubt with regard to applying these selection/exclusion criteria.

The complete texts of the selected articles were read, excluding any with patient ages below 18 years, or any that included patients without cardiovascular disease, even though the treatment applied or the parameters analyzed or objectives showed similarities to the other works selected for review.

Due to the scarcity of articles that met the inclusion criteria, studies were selected regardless of their methodology, the method of assigning treatment, sample size, follow-up period, or the clinical, biochemical or periodontal parameters assessed.

The clinical trials included for review were assessed using the Critical Appraisal Skills Program CASPe (Spanish version) (16) and the review itself was assessed using the Preferred Reporting Items for Systematic Reviews and Meta-Analyses (PRISMA) scale (17), with 20 items.

Pooled estimates from the studies were analyzed using a continuous random-effects model meta-analysis. The variable analyzed were changes produced in CRP and leukocytes under the following interventions: a) Oral hygiene instructions + Supragingival scaling + Root scaling and planing Vs. Oral hygiene instructions; b) Oral hygiene instructions + Supragingival scaling + Root scaling and planning Vs. Non treatment.

Forest plots were produced to graphically represent the difference in outcomes of CRP and leukocytes values. *P* = 0.05 was used as the level of signiﬁcance. Heterogeneity was assessed with x2 test and I2 test. The OpenMeta[analyst] tool was employed in the statistical analysis.

## Results

-Identified studies

Of the 192 articles initially located in the electronic search, 134 were discarded having read titles and abstracts, as they did not fulfill the selection criteria. The full texts of the 58 remaining articles were read, excluding two works as they focused on patients aged younger than 18 years, and 46 other works centered on patients without cardiovascular disease.

**Figure 1:**
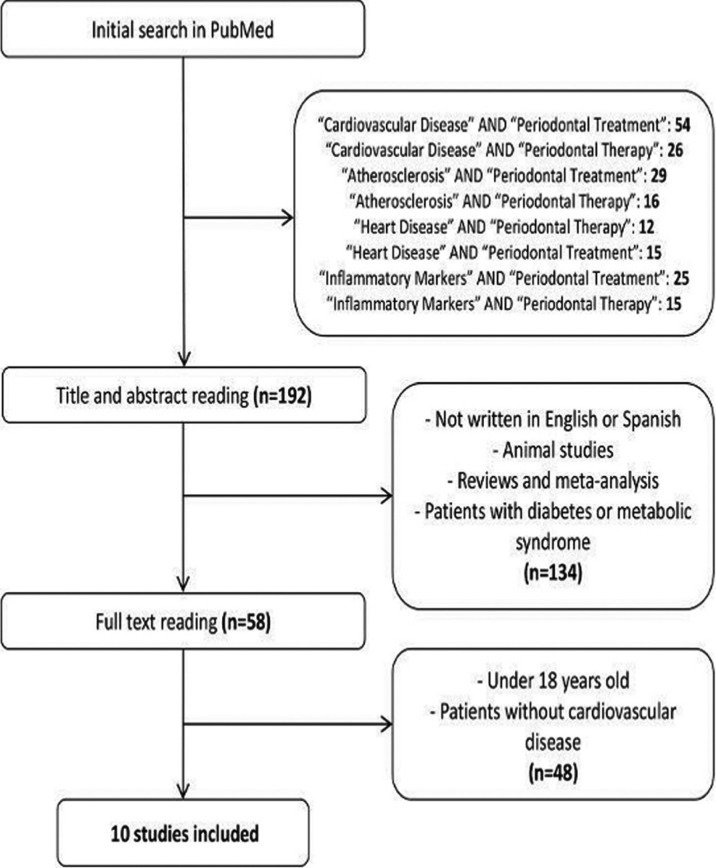
Flow diagram of selection process for articles: 10 trials were selected for analysis.

Following the selection process, the review analyzed a total of 10 clinical trials evaluating the effect of periodontal treatment on clinical and biochemical parameters involved in the development of ACVD and/or pertaining to patients diagnosed with ACVD aged over 18 years. The total population included in the studies was 669 patients (390 in intervention groups and 279 in control groups) (Fig. 1) ( Table 1,  Table 1 continue, Table 2).

**Table 1:**
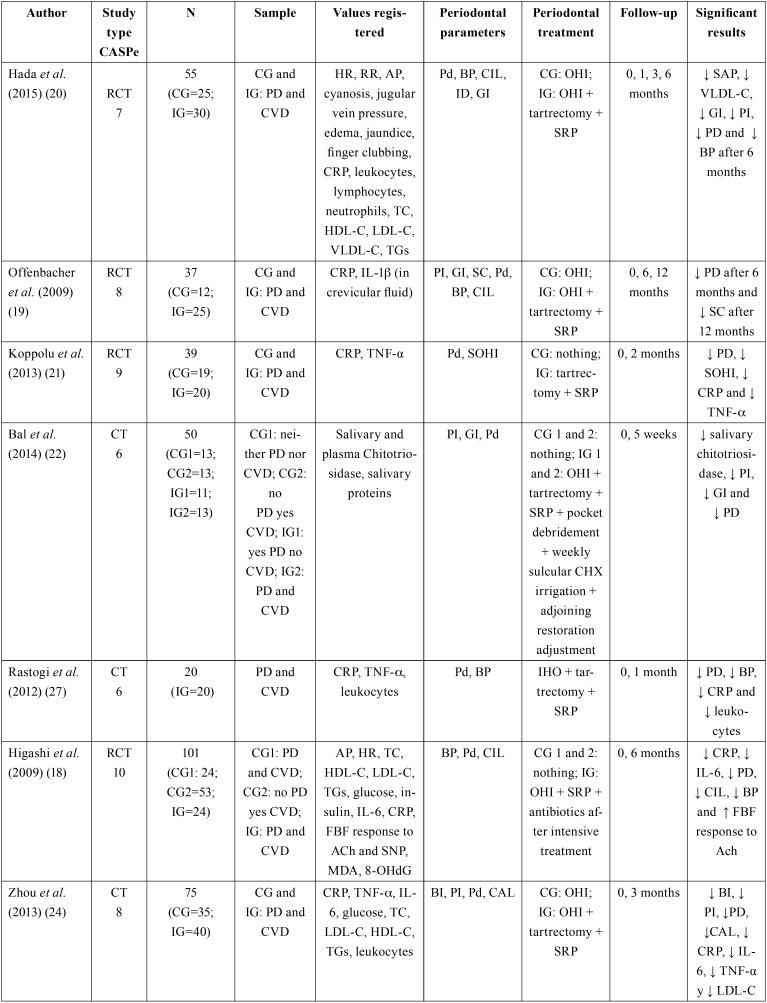
Summary of the clinical studies reviewed.

**Table 1 continue:**
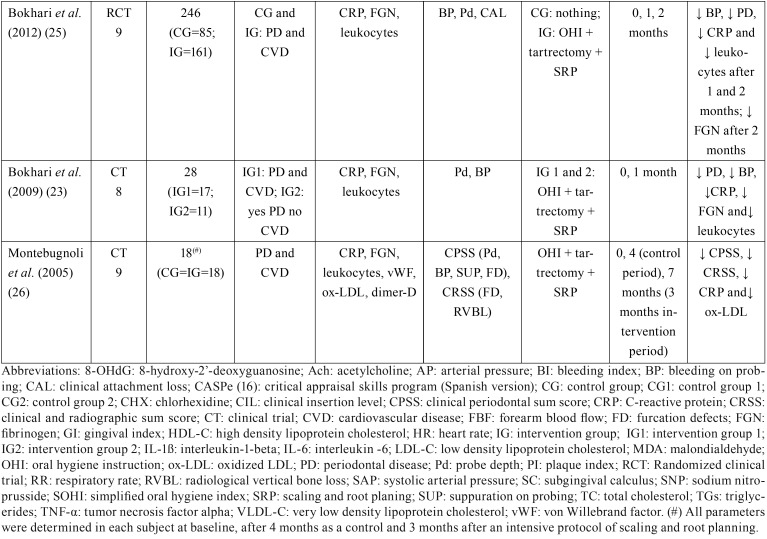
Summary of the clinical studies reviewed.

**Table 2:**
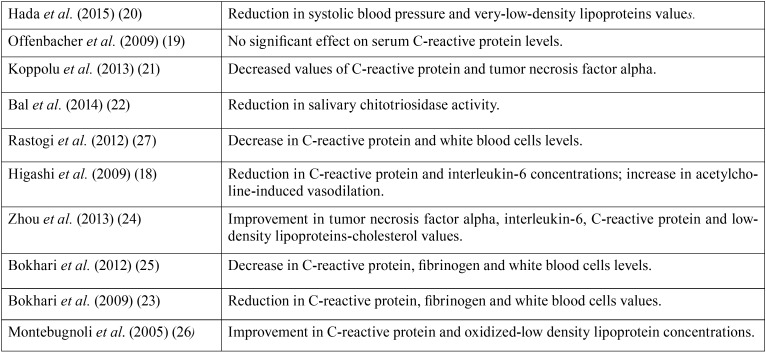
Summary of main results.

With the exception of two of these studies (18,19), all of them excluded patients with any acute or chronic conditions that could influence systemic inflammation and distort de results obtained.

-Periodontal treatment

The periodontal treatment performed in the studies reviewed consisted of mechanical debridement (n=390) (18-27) in all cases, complemented by oral hygiene instruction (OHI) in most of them (19,20,23). In other clinical trial periodontal pockets were reduced using a diamond bur (n=24) (22), and in another, antibiotic therapy was used in some cases depending on the intensity of the treatment applied (n=24) (18).

Selected articles did not show homogeneity in the diagnosis of periodontal disease. In the vast majority the term chronic periodontitis was used without specifying the degree, with exception of one in which patients with chronic gingivitis and mild to moderate periodontitis were included (20).

-ACVD diagnosis

The criteria defining cardiovascular pathology differed from study to study. In some cases, patients with previous histories of cardiovascular events (n=94) (20,21) were included, in others, patients with obstruction over 50% or over 70% in at least one artery (n=43) (22,23), or both (n=477) (18,19,24-26). In one article, subjects had been diagnosed with coronary artery pathology, although this was not specified in detail (n=20) (27).

-Periodontal parameters

The variables used to objectify improvement in periodontal status in all the studies reviewed were: probe depth, bleeding on probing, and gingival index. Clinical insertion level was measured in half the works (18-20,24,25). Follow-up periods ranged between one month and one year, with a mean of 15 weeks.

-Cardiovascular parameters

Regarding the cardiovascular risk parameters assessed, nine works analyzed CRP, (18-21,23-27) observing significant reductions in CRP levels after periodontal treatment (n=311) (18,21,23-27). TNF-ß was studied in three of the trials (21,24,27), finding a statistically significant reduction in two of them (n=60).(21,24) IL-6 was only measured in two works (18,24) observing significant reductions after periodontal treatment (n=64). IL-1α levels were only analyzed in a single work in crevicular fluid samples, but did not present any significant improvement after periodontal treatment (19).

As for lipid profiles, three works (18,20,24) analyzed plasma levels of total cholesterol, LDL-C, HDL-C and triglycerides. None of them observed significant changes in total cholesterol levels, HDL-C or triglycerides after periodontal treatment. Only one of them observed a significant reduction in LDL-C resulting from treatment (n=40) (24). A study conducted on 18 patients measured oxidized LDL, obtaining a significant reduction in levels after treatment (n=18) (26). VLDL cholesterol was only measured in one work,(20) also obtaining a significant reduction (n=30).

Total leukocyte levels were analyzed in six studies (20,23-27), observing significant reductions in three of them after periodontal treatment (n=209) (23,25,27). One of this clinical trials also measured lymphocyte and neutrophil levels individually, but did not observe any differences resulting from periodontal treatment (20). None of these six works included antibiotic therapy in periodontal treatment. Two of the three studies (23,25,26) that studied fibrogenin observed significant reductions (n=189) (23,25). Glucose levels were measured in two studiesb (18,24) but neither saw any changes. No significant changes were obtained regarding insulin levels after periodontal treatment (18).

-Other parameters

One work evaluated salivary and plasma chitotriosidase (enzyme produce by activated macrophages) and salivary proteins (22). After periodontal treatment, a significant reduction in the salivary enzyme was observed (n=24), put not in plasma or in the salivary proteins evaluated.

Another study (26) measured von Willebrand factor and fibrin D-dimer but did not observe significant differences after periodontal treatment.

Other parameters analyzed in the studies were malondialdehyde-modified LDL, 8-hydroxy-2’-deoxyguanosine, and forearm blood flow response to acetylcholine and sodium nitroprusside (18). After periodontal treatment, significant changes were observed in blood flow response to acetylcholine, considerably increasing vasodilation (n=24).

Heart rate and blood pressure were analyzed in two of the works (18,20). The only significant result was a reduction in systolic blood pressure observed in the first study (n=30) (20). This one also analyzed other clinical parameters (respiratory rate, the presence of cyanosis, jugular venous pressure, edema, jaundice, and soft tissue enlargement (clubbing) of the fingers) but no significant changes were produced in any of these parameters after treatment.

-CRP and leukocytes Meta-analysis

**Figure 2:**
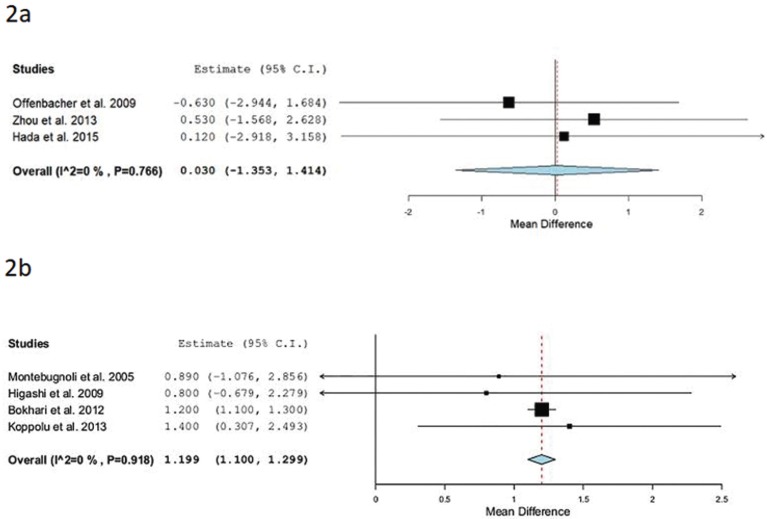
(a) Forest plot: studies evaluating CRP values before and after the administration of Oral hygiene instructions + Supragingival scaling + Scaling and root planing in the treatment group Vs. Oral hygiene instructions in the control group. (b) Forest plot: studies evaluating CRP values before and after the administration of Oral hygiene instructions + Supragingival scaling + Scaling and root planing in the treatment group Vs. Non treatment.

Separate meta-analysis was performed to analyze the mean differences of CRP between studies. Three studies (19,20,24) evaluated values of CRP before and after the administration of Oral hygiene instructions + Supragingival scaling + Scaling and root planning in the treatment group (n= 95) Vs. Oral hygiene instructions in the control group (n=72). The forest plot (Fig. 2a) shows a CRP mean difference of 0.03mg/l and a p value = 0.966 (95% CI: -1.353 to 1.414, heterogeneity I2 =0%, *P*=0.766).

Four studies (18,21,25,26) assessed values of CRP before and after the administration of Oral hygiene instructions + Supragingival scaling + Scaling and root planing in the treatment group (n= 223) Vs. Non treatment in the control group (n=146). The forest plot (Fig. 2b) shows a CRP mean difference of 1.199 mg/l and a *p* value = <0.001 (95% CI: 1.100 to 1.299, heterogeneity I2 =0%, *P*=0.918).

**Figure 3:**
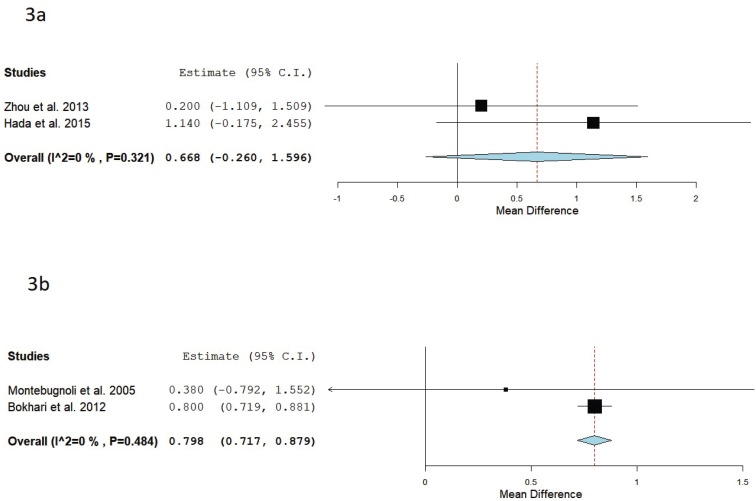
(a) Forest plot of studies evaluating leukocytes values before and after the administration of Oral hygiene instructions + Supragingival scaling + Root scaling and planning in the treatment group vs. Oral hygiene instructions in the control group.(b) Forest plot of studies evaluating leukocytes values before and after the administration of Oral hygiene instructions + Supragingival scaling + Root scaling and planning in the treatment group vs. Non treatment.

Also a meta-analysis was performed to analyze the mean differences of leukocytes between studies. Two studies (22,30) evaluated values of leukocytes before and after the administration of Oral hygiene instructions + Supragingival scaling + Root scaling and planning in the treatment group (n= 70) Vs. Oral hygiene instructions in the control group (n=60). The forest plot (Fig. 3a) shows a leukocytes mean difference of 0.67 g/l and a *p* value = 0.158 (95% CI: -0.260 to 1.596, heterogeneity I2 =0%, *P*=0.321).

Two studies31,32 assessed values of leukocytes before and after the administration of Oral hygiene instructions + Supragingival scaling + Root scaling and planning in the treatment group (n= 179) Vs. Non treatment in the control group (n=103). The forest plot (Fig. 3b) shows a leukocytes mean difference of 0.79 g/l and a *p* value = <0.001 (95% CI: 0.717 to 0.879, heterogeneity I2 =0%, *P*=0.484).

## Discussion

A 2017 Cochrane systematic review of randomized clinical trials conducted among patients with periodontal disease and ACVD antecedents (secondary prevention) or patients with periodontal disease but without ACVD antecedents (primary prevention), failed to find sufficient evidence to support or refute the possibility that periodontal treatment prevents or delays the recurrence of ACVD; no evidence for primary prevention of ACVD was found. However, this review only included a single clinical trial, in which part of the control group received non-surgical periodontal treatment outside the study within the follow-up period (28).

In the works analyzed in the present literature review, most of the studies that analyzed CRP levels after periodontal treatment (18,21,23-27) observed statistically significant reductions in this parameter with two exceptions (19,20). The periodontal treatment applied was the same in all six studies, but the different outcomes could be explained by the fact that in one work were included patients with advanced chronic gingivitis rather than severe chronic periodontitis, and that baseline values were significantly different between the intervention and control groups (20). In another clinical trial, control group patients were permitted to undergo periodontal treatment independently of the study, so that the real periodontal attention received by these patients was unknown, a factor that could exert some influence in the absence of differences in periodontal status between the groups after treatment (19).

In two trials conducted with samples of 39 (21) and 75 (24) subjects with follow-ups of 2 and 3 months respectively, the intervention groups underwent mechanical rather than surgical debridement, obtaining statistically significant reductions (*p*<0.05) in TFN-α values after periodontal treatment. But a study that applied the same treatment to a sample of 20 patients with a 1-month follow-up, did not observe significant changes (*p*>0.05) in this parameter (27). This could be due to the small sample size or the brief follow-up period.

Two of the works that studied IL-6 obtained significant reductions (*p*<0.05) in levels after periodontal treatment (18,24). So, it may be deduced that mechanical debridement reduces serum levels of this soluble protein in this type of patient. The same did not occur with IL-1ß, registered in a sample of 37 patients with a 12-month follow-up (19). However, this study obtained values proceeding from crevicular fluid, which were not correlated to serum levels.

Mechanical debridement of periodontal tissues would appear unrelated to improvements in total cholesterol, HDL-C and triglyceride levels, as none of the three trials (18,20,24) that analyzed these parameters observed significant changes (*p*>0.05). Nevertheless, it seems that periodontal treatment could be related to LDL, VLDL and oxidized LDL levels, as these values decreased significantly in three works (*p*<0.05, *p*<0.05, *p*<0.01 respectively) (20,24,26). If these parameters can be improved by periodontal treatment, this could constitute a valuable means of ACVD prevention, as these are some of the main components of atheromatous plaque (6).

Improving periodontal status could also be associated with a reduction in total leukocytes. Half of the trials that analyzed this parameter obtained significant reductions (*p*<0.05) in values (23,24,27), although the other three studies that looked into this parameter (20,24,26) did not observe significant changes. Studies with follow-up periods of 1 or 2 months observed significant changes (23,25,27), while the others had longer follow-up periods. This suggests that perhaps leukocyte levels only respond to periodontal treatment for a limited period. These results coincide with the meta-analysis performed where a significant difference was found when oral hygiene instructions + supragingival scaling + root scaling and planning were applied compared to non-treatment.

Fibrinogen levels appear to act similarly, as significant reductions (*p*<0.05) were observed in two trials with shorter follow-up periods of between 1 and 2 months after periodontal treatment (23,25). But in a study with a 3-month follow-up (in which no intermediate measurements were performed) no changes were produced (26). This could be explained by the possibility that fibrinogen responded to mechanical debridement for a brief period, or perhaps due to reductions in gingival bleeding after treatment.

Glycemia was analyzed in two works (18,24) with follow-up periods of 6 and 3 months respectively. Neither of these observed significant reductions in glycemia (*p*>0.05) after periodontal treatment, which could be due to this parameter’s poor response to mechanical debridement or to improvements in periodontal status, or to the possibility that the effect only continued for a limited period. The first of these two studies also registered insulin levels with the same results.

Nor was periodontal treatment seen to reduce plasma chitotriosidase or salivary proteins, although salivary chitotriosidase levels were reduced (*p*=0.005) (22). Improvements in periodontal status could be associated with the reestablishment of some salivary parameters, which do not extend to systemic improvement.

Mechanical debridement did not appear to have any effect on von Willebrand factor or on fibrin D-dimer, although further trials are necessary to confirm this, as the present review only found a single study that investigated these parameters (26).

Blood flow response to acetylcholine increased considerably (*p*<0.001) after periodontal treatment, improving the endothelial dysfunction typical of periodontal patients (18). However, no changes in malondialdehyde-modified LDL, 8-hydroxy-2’-deoxyguanosine, or forearm blood flow response to sodium nitroprusside were observed. As this analysis took place at the end of a 6-month follow-up, there could have been some short-term improvement that was not registered.

Only two of the works reviewed considered non-analytic parameters (18,20), and the only parameter to show a statistically significant improvement (*p*<0.05) was systolic blood pressure (20). Further studies with longer follow-up periods would be needed to determine whether periodontal treatment improves clinical parameters of cardiovascular patients beyond the biochemical parameters analyzed in most of the trials conducted to date.

In agreement with the findings of the present review, a meta-analysis published in 2014 concluded that periodontal treatment improves endothelial function and the atherosclerotic profile of periodontal patients with ACVD and/or diabetes (29). Similarly, an earlier 2013 systematic review affirmed that periodontal treatment produces a progressive reduction in systemic inflammation and improvements in endothelial function. However, there is no evidence that these changes have any long-term effect on cardiovascular risk in patients with periodontitis (3).

Lastly, another meta-analysis that focused on CRP values alone concluded that non-surgical periodontal treatment reduced serum levels of this protein (30). This agrees with the results obtained in the meta-analysis performed in the present review, where a decreased in CRP values was noted to be statistically significant when patients were submitted to non-surgical periodontal treatment in contrast to receiving no treatment at all.

Even the positive results obtained, considering the limitations discussed throughout the study, more homogeneous clinical trials, with larger samples, more detailed methodology and longer follow-up periods are needed in order to generalize these findings.

In conclusion, periodontal treatment can improve some biochemical parameters involved in the development of ACVD, including CRP, TNF-α, IL-6, fibrinogen, leukocytes, oxidized LDL, and VLDL-C. The non-analytic parameters investigated did not respond to periodontal treatment. Further clinical studies with longer follow-up periods and regular periodic analyses are necessary to establish the effect of non-surgical periodontal treatment on patients with ACVD.
